# Effects of mechanical repetitive load on bone quality around implants in rat maxillae

**DOI:** 10.1371/journal.pone.0189893

**Published:** 2017-12-15

**Authors:** Yusuke Uto, Shinichiro Kuroshima, Takayoshi Nakano, Takuya Ishimoto, Nao Inaba, Yusuke Uchida, Takashi Sawase

**Affiliations:** 1 Department of Applied Prosthodontics, Graduate School of Biomedical Sciences, Nagasaki University, Nagasaki, Japan; 2 Division of Materials and Manufacturing Science, Graduate School of Engineering, Osaka University, Yamadaoka, Suita-city, Osaka, Japan; Indiana University Purdue University at Indianapolis, UNITED STATES

## Abstract

Greater understanding and acceptance of the new concept “bone quality”, which was proposed by the National Institutes of Health and is based on bone cells and collagen fibers, are required. The novel protein Semaphorin3A (Sema3A) is associated with osteoprotection by regulating bone cells. The aims of this study were to investigate the effects of mechanical loads on Sema3A production and bone quality based on bone cells and collagen fibers around implants in rat maxillae. Grade IV-titanium threaded implants were placed at 4 weeks post-extraction in maxillary first molars. Implants received mechanical loads (10 N, 3 Hz for 1800 cycles, 2 days/week) for 5 weeks from 3 weeks post-implant placement to minimize the effects of wound healing processes by implant placement. Bone structures, bone mineral density (BMD), Sema3A production and bone quality based on bone cells and collagen fibers were analyzed using microcomputed tomography, histomorphometry, immunohistomorphometry, polarized light microscopy and birefringence measurement system inside of the first and second thread (designated as thread A and B, respectively), as mechanical stresses are concentrated and differently distributed on the first two threads from the implant neck. Mechanical load significantly increased BMD, but not bone volume around implants. Inside thread B, but not thread A, mechanical load significantly accelerated Sema3A production with increased number of osteoblasts and osteocytes, and enhanced production of both type I and III collagen. Moreover, mechanical load also significantly induced preferential alignment of collagen fibers in the lower flank of thread B. These data demonstrate that mechanical load has different effects on Sema3A production and bone quality based on bone cells and collagen fibers between the inside threads of A and B. Mechanical load-induced Sema3A production may be differentially regulated by the type of bone structure or distinct stress distribution, resulting in control of bone quality around implants in jaw bones.

## Introduction

Dental implants are constantly subject to mechanical loads such as masticatory and swallowing forces via superstructures. In particular, mechanical stresses concentrate on the marginal bone around dental implants [1], suggesting that the maintenance of marginal bone levels for the long-term is important for successful clinical outcomes. To determine the effects of mechanical stimulation on bone around dental implants, finite element analyses have been mainly used [2–4]. Moreover, some studies have reported the effects on the jaw bone around implants using occlusal forces [5, 6]. However, occlusal forces are unstable, as the magnitude, frequency, cycles and direction of the loads can change irregularly. Thus, animal studies using controlled-mechanical stimuli are absolutely required to clarify the net effect of mechanical loads on jaw bone reactions around implants.

Prior to 2000, bone strength was synonymous with bone mineral density (BMD). The National Institutes of Health (NIH) has since proposed a new clinical parameter; “bone quality” [7]. Bone quality, which is completely independent of BMD, comprises bone architecture, bone turnover, bone mineralization and micro-damage accumulation [7, 8]. Moreover, bone cells such as osteoblasts and osteocytes, and characteristics of collagen fibers, including type and alignment, are thought to be the determinant factors of bone quality [9]. In the intact mandible, basically, orientation of collagen fibers and the related biological apatite crystals uni-directionally aligns along the mesiodistal axis; however, the direction of maximum orientation has been demonstrated to change locally along the biting direction just beneath the teeth, indicating that biting stress is effectively and continuously transmitted from teeth to the host bone in a normal tooth-mandible system [10]. Thus, the appropriate orientation of calcified collagen fibers should be obtained in the regenerative bone surrounding dental implants. Our recent studies using rabbit tibiae demonstrated that mechanical repetitive load along the long axis of implants improves bone quality around dental implants orthogonally placed to the long axis of tibiae, with the development of osteocyte networks and preferential alignment of collagen fibers [11–13]. In particular, bone quality inside the grooves of the implant neck are improved by mechanical repetitive load using rabbit long bones [11]. However, bone structures of rabbit tibiae are entirely different from jaw bone structures, specifically with regard to the small amount of trabecular bone, suggesting that rabbits are an undesirable species to investigate bone reactions to mechanical loads around dental implants [14]. Moreover, stress distributions differ between jaw and long bones around implants, due to the differences in bone structure and density [15], and loading direction (parallel vs. orthogonal along the long axis of implants). Therefore, the use of jaw bones, but not long bones, is strongly recommended to clarify the net effect of mechanical load on bone around dental implants.

Some mechanisms by which bone reacts to mechanical stresses have been demonstrated. Mechanical loads are mainly converted into mechanical stimuli such as fluid shear stress, hydrostatic pressure, and direct cell deformation. Mechanical stimuli induce matrix deformation around osteocytes and cell processes, promoting the production of signaling molecules by osteocytes [16]. Hence, osteocytes play an important role as mechanosensors. Recently, our study indicated that osteocytes determine the normalization of collagen alignments when osteocytes properly respond to mechanical stimuli [17]. We also demonstrated that collagen fibers produced by osteoblasts preferentially orient along the osteoblast-elongated direction, suggesting that osteoblast alignment determines extracellular matrix orientation [18]. Impaired cross-linking in collagenous matrix affects osteoblast differentiation [19]. On the other hand, osteoclast functions, including resorption of extracellular matrix, have been shown to be controlled via regulation of osteoclastogenesis in response to several mechanical stimuli [20]. Thus, bone cells, especially osteoblasts and osteocytes, are interdependently linked with collagen fibers under loaded-conditions. However, scientific evidence for bone quality based on bone cells and collagen fibers around implants in jaw bone remains elusive. Thus, the investigation of bone cells with collagen fibers is required to elucidate bone quality around implants under loaded conditions.

Semaphorin3A (Sema3A), a novel secreted protein, has mainly been studied in the nervous system, oncology (e.g., multiple myeloma and tumor progression) and several types of autoimmune disease (e.g., rheumatoid arthritis and systemic lupus erythematosus) [21–23]. Recently, Sema3A, which is expressed by osteoblasts and osteoclast-like multinuclear cells, has been demonstrated to play an important role in osteoblast and osteoclast regulation, also known as osteoprotection [23, 24]. The latest study reported that Sema3A mRNA levels are upregulated by centrifugation stress in MC3T3-E1 cells [25]. However, Sema3A in bone tissue around implants is not fully understood under non-loaded and loaded-conditions.

Accordingly, our hypotheses are that: (1) mechanical loads improve bone quality around implants, based on bone cells and collagen fibers; and (2) osteoprotection occurs in bone around implants via mechanical load-induced Sema3A production. To evaluate the net effect of mechanical load on “bone quality” around implants, the jaw bone, but not long bones, should be used. The aims of this study were: (1) to clarify the effects of mechanical repetitive load on bone quality based on bone cells and collagen fibers around implants in rat maxillae, and (2) to investigate the effects of mechanical load on Sema3A production in bone around implants in rat maxillae.

## Materials and methods

### Animals and surgical procedures

**Fig 1:**
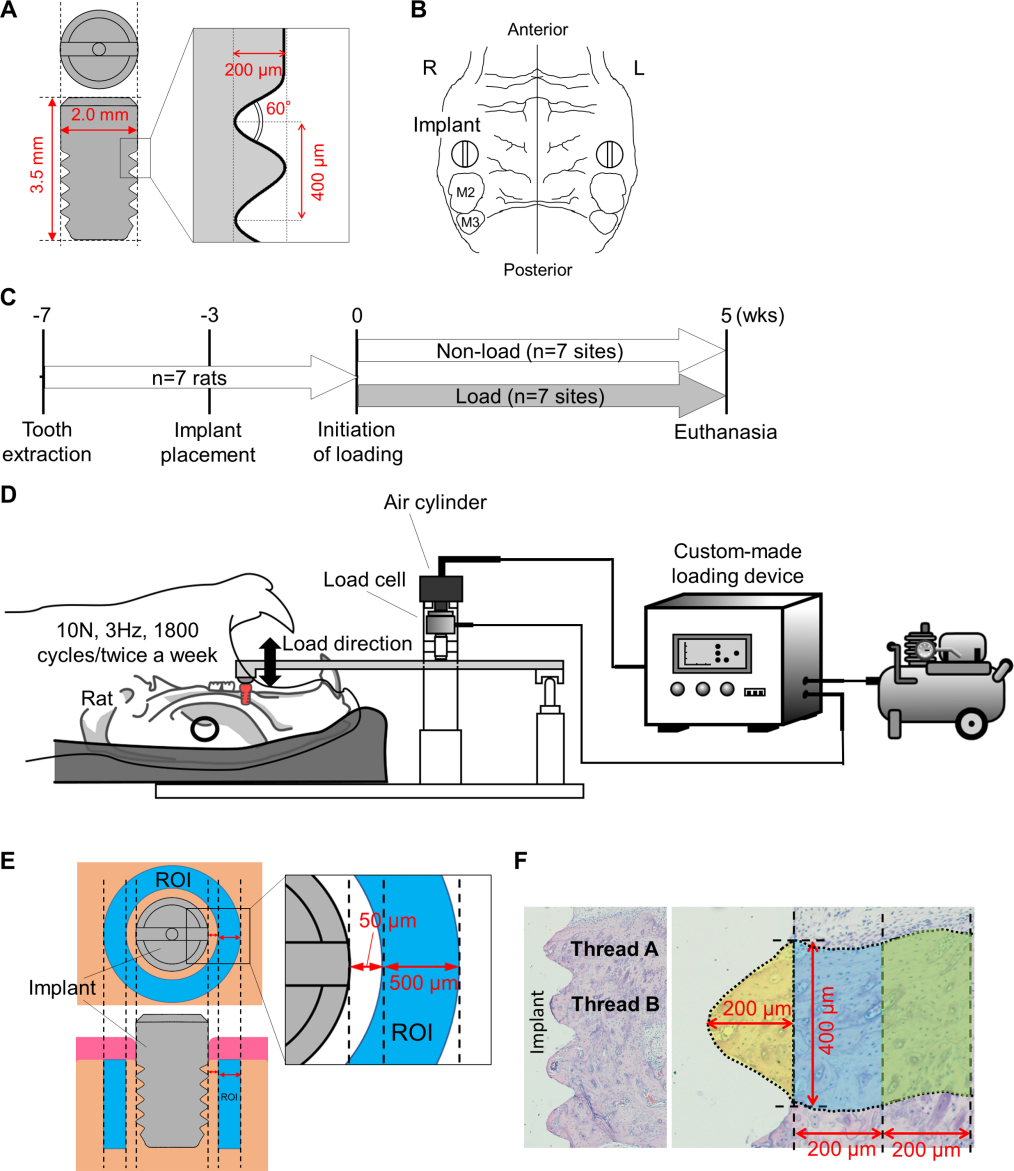
Experimental design. (A) Scheme of titanium implant. Length and diameter of implants, and pitch, height and angle of thread were presented. (B) Implant placement was performed after extraction of both maxillary first molars (M2 and M3 indicate second and third molar, respectively). (C) Experimental schedule. Seven rats were used. A randomly selected implant per rat received mechanical loads (n = 7 sites). Remaining implants received no mechanical loads (n = 7 sites, control). (D) Mechanical repetitive load (10N, 3Hz, and 1800 cycles) was applied twice a week for 5 weeks at 3 weeks post-implant placement. (E) Region of interest (ROI; aqua area) for micro computed tomography (microCT) analysis. ROI did not include the area from the implant surface to 50μm away from implants to avoid titanium metal artifacts. (F) Areas of interest (AOIs; yellowish area, aqua area and yellow-green area) for histological analyses. Analyses were performed on the inside and outside areas of the first thread (designated as thread A) and the second thread (designated as thread B).

Seven female Wistar rats (9-month-old) were purchased (Kyudo Co., Ltd, Saga, Japan). Grade IV-titanium threaded implants with 2.0 mm in diameter, 3.5 mm in length, and 400 μm and 200 μm thread pitch and height, respectively were obtained (Kyocera Co., Kyoto, Japan; Fig 1A). Thread angles were 60° along the long axis of implants (Fig 1A). To compensate for complete wound healing after tooth extraction [26], implants were placed under general and local anesthesia at 4 weeks after the removal of both maxillary first molars (Fig 1B). Randomly selected implants from each rat were stimulated at 10 N with a frequency of 3 Hz for 1800 cycles, 2 days/week for 5 weeks using a custom-made loading device (Higuchi Co., Nagasaki, Japan) to mimic rat mastication [27, 28] (loading group; n = 7 sites) (Fig 1C). To investigate the net effect of mechanical load on bone around implants, mechanical repetitive load was initiated at 3 weeks post-implant placement, as implants are fully integrated into bone at 3 weeks post-implantation in rat maxillae [29]. Load direction was along with the long axis of implants. Cranial anchorage was performed to avoid the attenuation of mechanical stimuli (Fig 1D). Implants on the remaining side did not receive any mechanical stimuli (control group; n = 7 sites). Animal care and experimental procedures were performed in accordance with the Guidelines for Animal Experimentation of Nagasaki University, with approval from the Ethics Committee for Animal Research (Approval number: 1306141071–4).

### Micro computed tomography (microCT)

All experimental rats survived uneventfully before euthanasia. Hence, seven rats were euthanized after application of the last mechanical loads. Maxillae were dissected and fixed in 10% formalin, and stored at 4°C before use. Hard tissue around implants was scanned by microCT at 20-μm voxel resolution with an energy level of 90 kV (R_mCT2; Rigaku Co., Tokyo, Japan). Bone around implants was segmented and reconstructed by semi-manual contouring and was analyzed with a TRI/3D-Bon (Ratoc System Engineering, Tokyo, Japan). A region of interest (ROI) was defined as the region cross-sectionally surrounding between 50 μm and 550 μm away from the implant surface in order to avoid metal artifacts [30], and longitudinally surrounding the implant neck to the top of implant (Fig 1E). Bone parameters such as bone volume fraction [BV/TV (%)], trabecular number [Tb.N (1/mm)], trabecular thickness [Tb.Th (mm)], trabecular separation [Tb.Sp (mm)] and BMD [BMD (mg/cc)] were evaluated by the direct-measure technique [26, 31]

### Histomorphometric and immunohistomorphometric analyses

Just after microCT scanning, bone blocks including implants were demineralized in 10% ethylenediaminetetraacetic acid for 21 days at 4°C. Implants were removed by inverse rotation with great care after demineralization, and were then paraffin-embedded, and sectioned at 5 μm-thickness. Hematoxylin and eosin (H-E) staining was performed using a standard staining protocol to evaluate bone formation around implants and osteocyte numbers using light microscopy (Axio Scope A1; Zeiss, Oberkochen, Germany). Picrosirius red staining and tartrate-resistant acid phosphatase (TRAP) staining were carried out using commercial kits in accordance with manufacturer’s instructions (Direct Red 80 and 386A, respectively; Sigma Aldrich, St. Louis, MO) to visualize collagen fibers and osteoclasts, respectively. Type I and III collagen fibers were separately evaluated by polarized light microscopy (Axio Lab. A1; Zeiss). Immunostaining was also conducted to detect osteoblasts and Sema3A. Moreover, sections were incubated using primary antibodies overnight at 4°C. Rabbit anti-Rat Runx2 polyclonal antibody (ab23981; Abcam, Cambridge, MA) at 1:800 dilution for the detection of osteoblasts, and Rabbit anti-Rat Semaphorin3A polyclonal antibody (ab23393; Abcam) at 1:400 dilution were used as primary antibodies. Endogenous peroxidase activity was blocked with 3.0% hydrogen peroxidase. Goat anti-Rabbit immunoglobulinG conjugated to horseradish peroxidase (HRP) were used as secondary antibodies for 1 hour at room temperature. Proteins were developed with 3,3-diaminobenzidine. Sections were counterstained with Hematoxylin and mounted. Stained sections were photomicrographed using light microscopy (Axio Scope A1; Zeiss). Histomorphometric analyses were performed using software (ZEN2; Zeiss) and NIH imageJ (version 1.47; NIH, Bethesda, MD). Areas of interest (AOIs) were defined as the inside area of the first thread (designated as thread A) and second thread (designated as thread B) adjacent to the implant neck (AOI: 200 μm x 400 μm) (Fig 1F), as mechanical stresses were differentially distributed on the first two threads in the implant neck [1]. The following parameters were quantitatively evaluated: 1) osteocyte numbers in each AOI inside threads A and B [osteocyte density (#/mm^2^)]; 2) osteoblast number [32]: Runx2^+^ osteoblast numbers per linear bone perimeter [N.Ob/BS (#/mm)]; 3) osteoclast number [32]: osteoclast numbers per tissue area [N.Oc/T.A (#/mm^2^)]; 4) number of Sema3A per tissue area [Sema3A/T.A (#/mm^2^)]; and 5) type I and III collagen fraction: ratio of collagen fibers occupying each AOI [type I collagen area fraction (%) and type III collagen area fraction (%), respectively]. Measurement of collagen was performed both inside threads and outside threads at 0–200 μm and 200–400 μm away from implants.

### Assessment of preferential alignment of collagen fibers using birefringence measurement system

To evaluate the degree of preferential alignment of collagen fibers around implants, a birefringence measurement system WPA-micro (Photonic Lattice, Miyagi, Japan) attached to an upright microscope (Olympus, Tokyo, Japan) was used, as described previously [17]. Briefly, non-stained deparaffinized sections were imaged with a 20x or 50x objective. Data were acquired as the average of fifty images, with three settings of circularly polarized monochromatic light (laser wavelengths: 523, 543 and 575 nm) for each image. Orientation of the polarization axis with the greater index of refraction (slow axis) of specimens was analyzed with WPA-VIEW software (version 2.4.2.9, Photonic Lattice). Measurement of collagen alignment was performed both inside and outside threads at 0–200 μm and 200–400 μm away from implants.

### Statistical analyses

All statistical analyses were performed in a blind manner. The Shapiro-Wilk test was performed for normality. Student’s *t*-test and the Mann Whitney *U*-test were used for parametric and non-parametric data, respectively. In the comparison of three groups, one-way analysis of variance (ANOVA) and the Kruskal-Wallis test were used for parametric and non-parametric data, respectively. All statistical analyses were conducted using Systat 13 (Systat Software, Chicago, IL). All data are presented as means ± SEM.

## Results

### Effects of mechanical repetitive load on bone architecture

**Fig 2:**
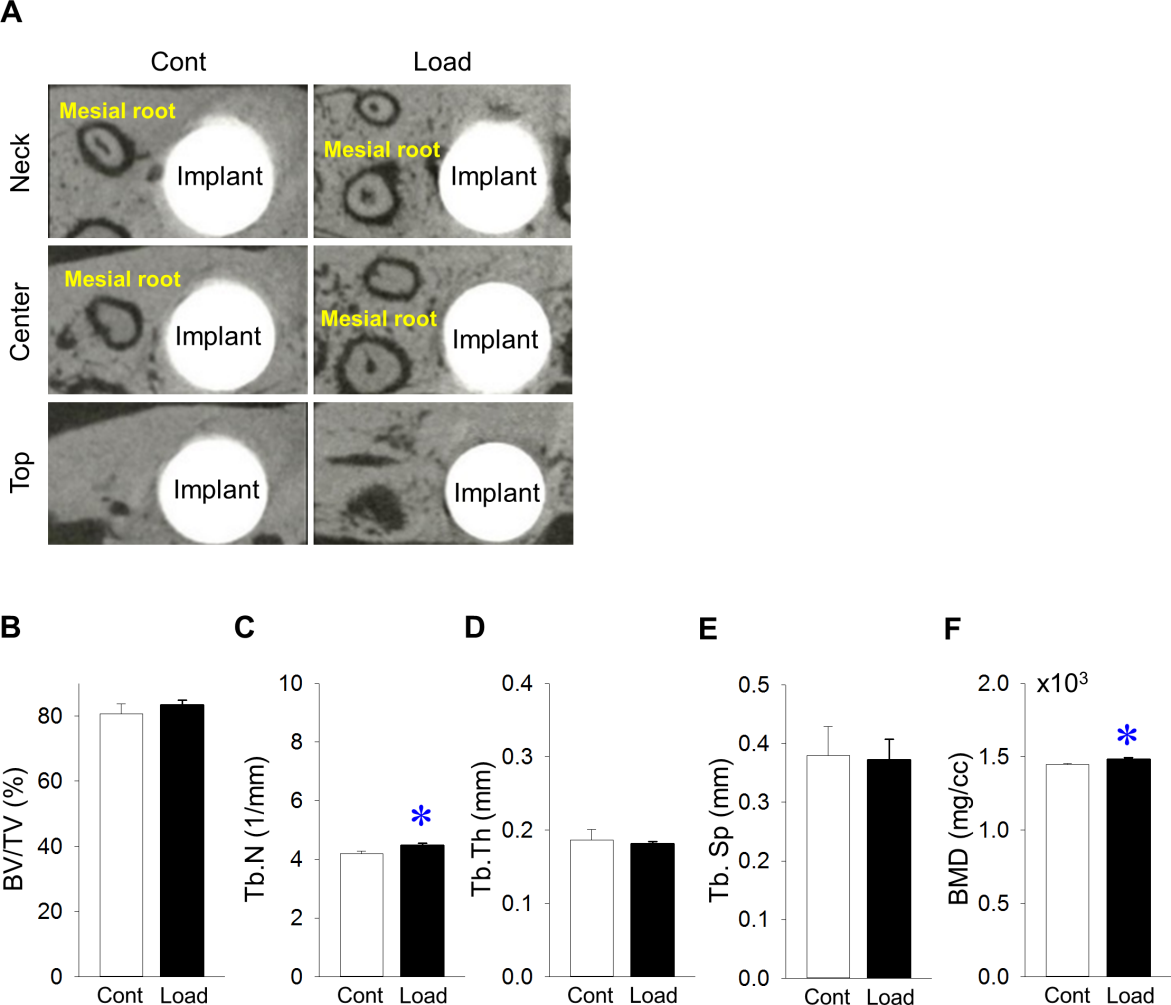
Effects of mechanical repetitive load on bone around implants. (A) Representative transverse microCT images at the neck, center and top of the implant. (B) Bone volume/tissue volume (BV/TV) around implants did not change between groups (Mesial root: mesial root of maxillary second molars). (C) Trabecular number (Tb.N) was significantly larger in the loading vs. control groups. (D) Trabecular thickness (Tb.Th) was the same between groups. (E) Trabecular separation (Tb.Sp) was similar between groups. (F) Bone mineral density (BMD) around implants increased significantly in the loading vs. control groups. (n = 7, *p<0.05).

Representative microCT images of bone at the neck, center and top of the implants (Fig 2A). Bone volume (BV/TV) around implants was the same, irrespective of mechanical load (Fig 2B). Tb.N in the loading group was significantly larger than that in the control group (Fig 2C). Tb.Th and Tb.Sp were similar, regardless of mechanical load (Fig 2D and 2E, respectively). BMD around implants in the loading group was significantly larger than that in the control group (Fig 2F).

### Effects of mechanical repetitive load on bone inside threads A and B

**Fig 3:**
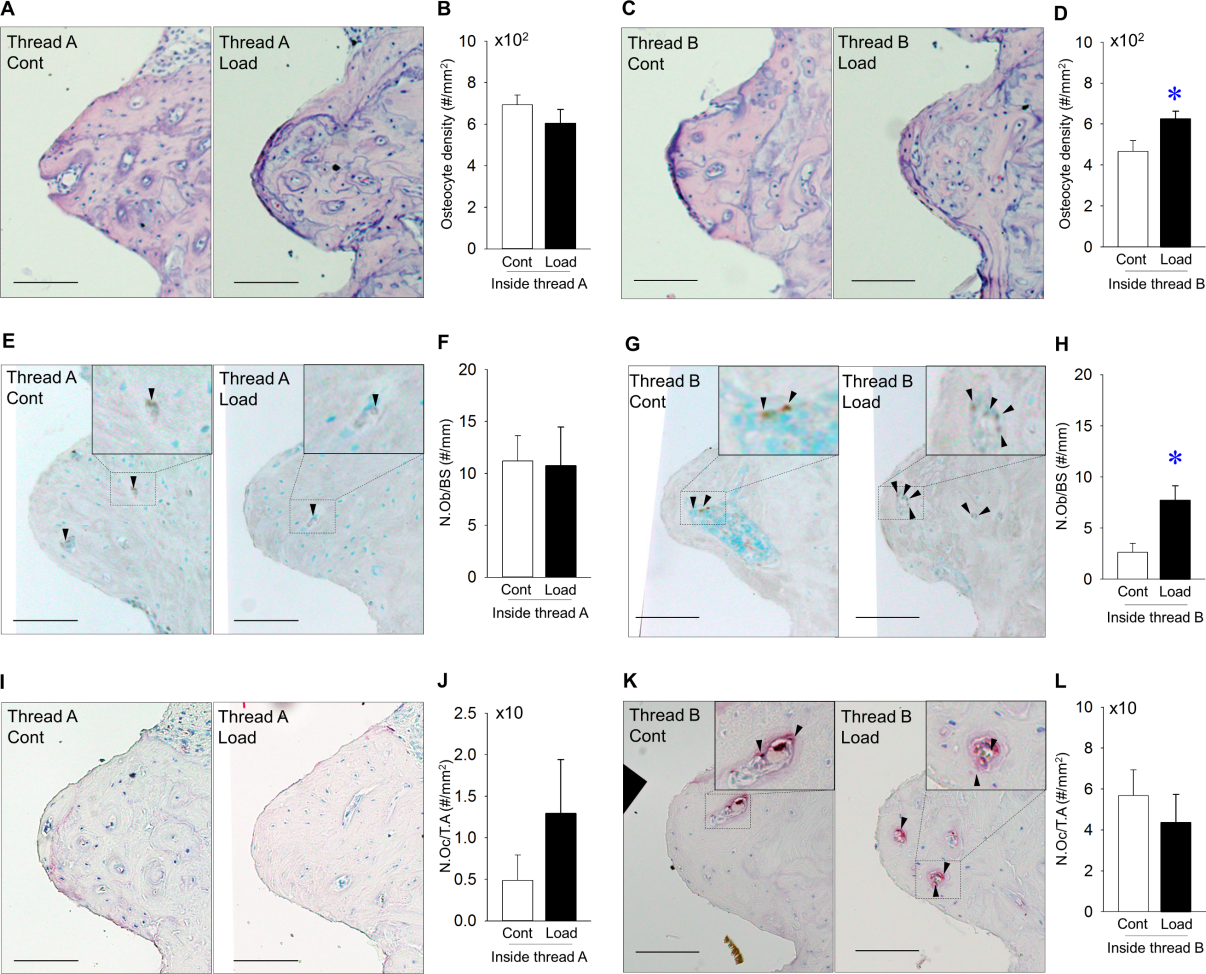
**Effects of mechanical repetitive load on bone inside threads A and B** (A) Representative images of H-E-stained sections (Bar: 100 μm). (B) Osteocyte density was the same inside thread A in the loading vs. control groups. (C) Representative images of H-E-stained sections (Bar: 100 μm). (D) Osteocyte density was significantly higher inside thread B in the loading vs. control groups. (E) Representative images of Runx2 stained-sections (Bar: 100μm; Arrowheads indicate Runx2^+^ osteoblasts). (F) Osteoblast number (N.Ob/BS) was the same inside thread A in the loading vs. control groups. (G) Representative images of Runx2-stained sections (Bar: 100μm; arrowheads indicate Runx2^+^ osteoblasts). (H) N.Ob/BS was significantly increased inside thread B in the loading vs. control group. (I) Representative images of TRAP-stained sections (Bar: 100 μm; Arrowheads indicate TRAP^+^ osteoclasts). (J) Osteoclast number (N.Oc/T.A) did not change inside thread A between groups. (K) Representative images of TRAP-stained sections (Bar: 100 μm; arrowheads indicate TRAP^+^ osteoclasts). (L) N.Oc/T.A was the same between groups. (n = 7 each, *p<0.05).

Representative H-E-stained images inside area of thread A. No infection was observed (Fig 3A). Osteocyte density was the same inside thread A, regardless of mechanical load (Fig 3B). Representative H-E-stained images of the inside area of thread B. No infection was noted (Fig 3C). Mechanical repetitive loads significantly increased osteocyte density inside thread B (Fig 3D). Representative Runx-2-stained images of the inside area of thread A (Fig 3E). Mechanical repetitive load did not change the number of osteoblasts inside thread A (Fig 3F). Representative Runx-2-stained images of the inside area of thread B (Fig 3G). Mechanical repetitive loads significantly increased the number of osteoblasts inside thread B (Fig 3H). Representative TRAP-stained images of the inside area of thread A. (Fig 3I). The number of osteoclasts was almost the same inside thread A, irrespective of mechanical load (Fig 3J). Representative TRAP-stained images of the inside area of thread B (Fig 3K). The number of osteoclasts inside thread B was similar, irrespective of mechanical repetitive load (Fig 3L).

### Effects of mechanical repetitive loads on Sema3A production inside threads A and B

**Fig 4:**
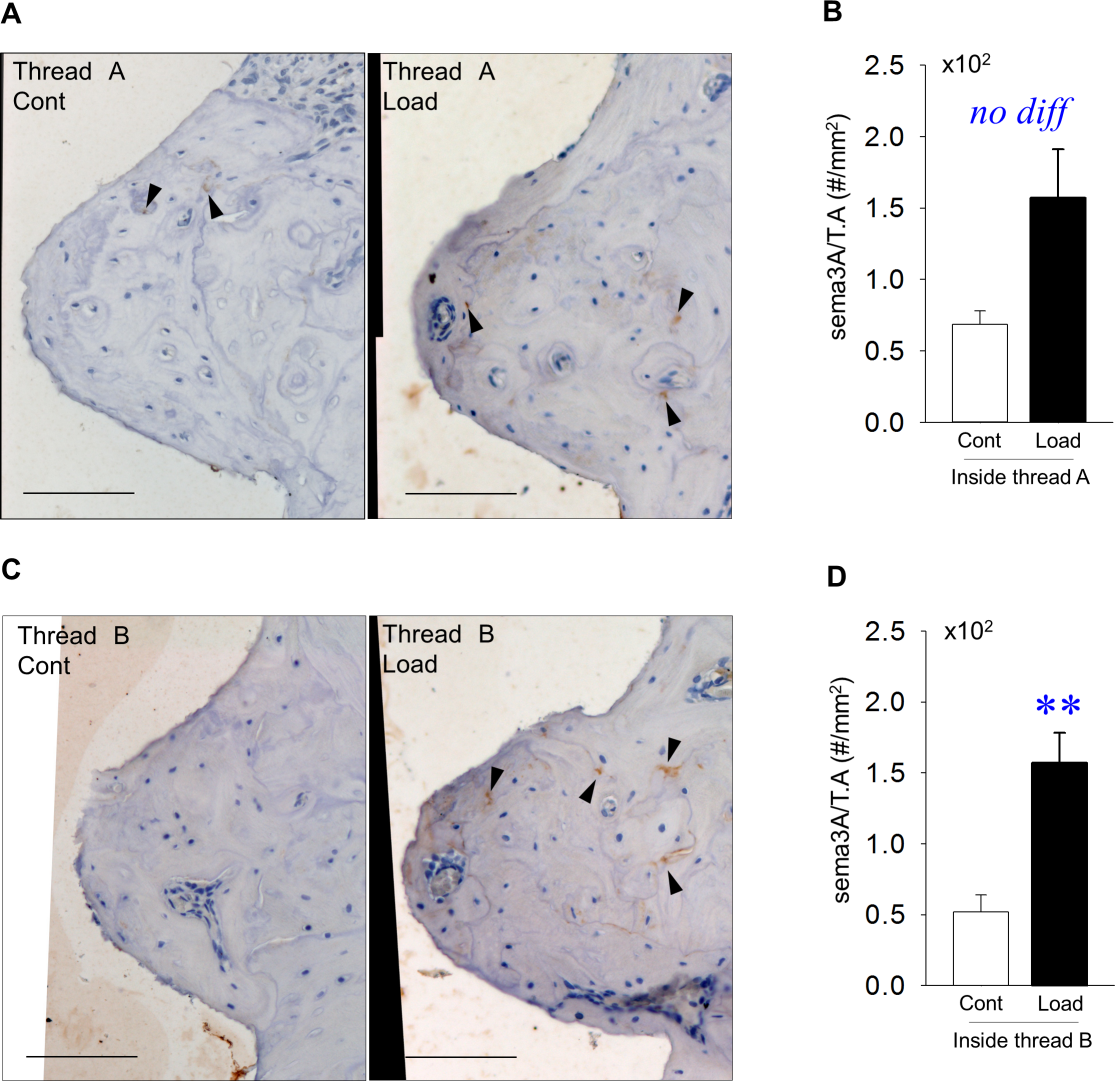
**Production of Sema3A inside threads A and B** (A) Representative images of Sema3A-stained sections of the inside area of thread A (Bar: 100 μm). (B) Sema3A levels were similar between groups. (C) Representative images of Sema3A-stained sections of the inside area of thread B (Bar: 100μm). (D) Sema3A levels were significantly higher in the loading vs. control group. (arrowhead: Sema3A, n = 7, *p<0.05).

Representative Sema3A-stained images of the inside area of thread A (Fig 4A). Sema3A levels inside thread A were the same, regardless of mechanical repetitive load (Fig 4B). Representative Sema3A-stained images of the inside area of thread B (Fig 4C). Mechanical repetitive loads significantly increased Sema3A levels inside thread B (Fig 4D).

### Effects of mechanical repetitive loads on collagen fibers inside and outside areas of threads A and B

**Fig 5:**
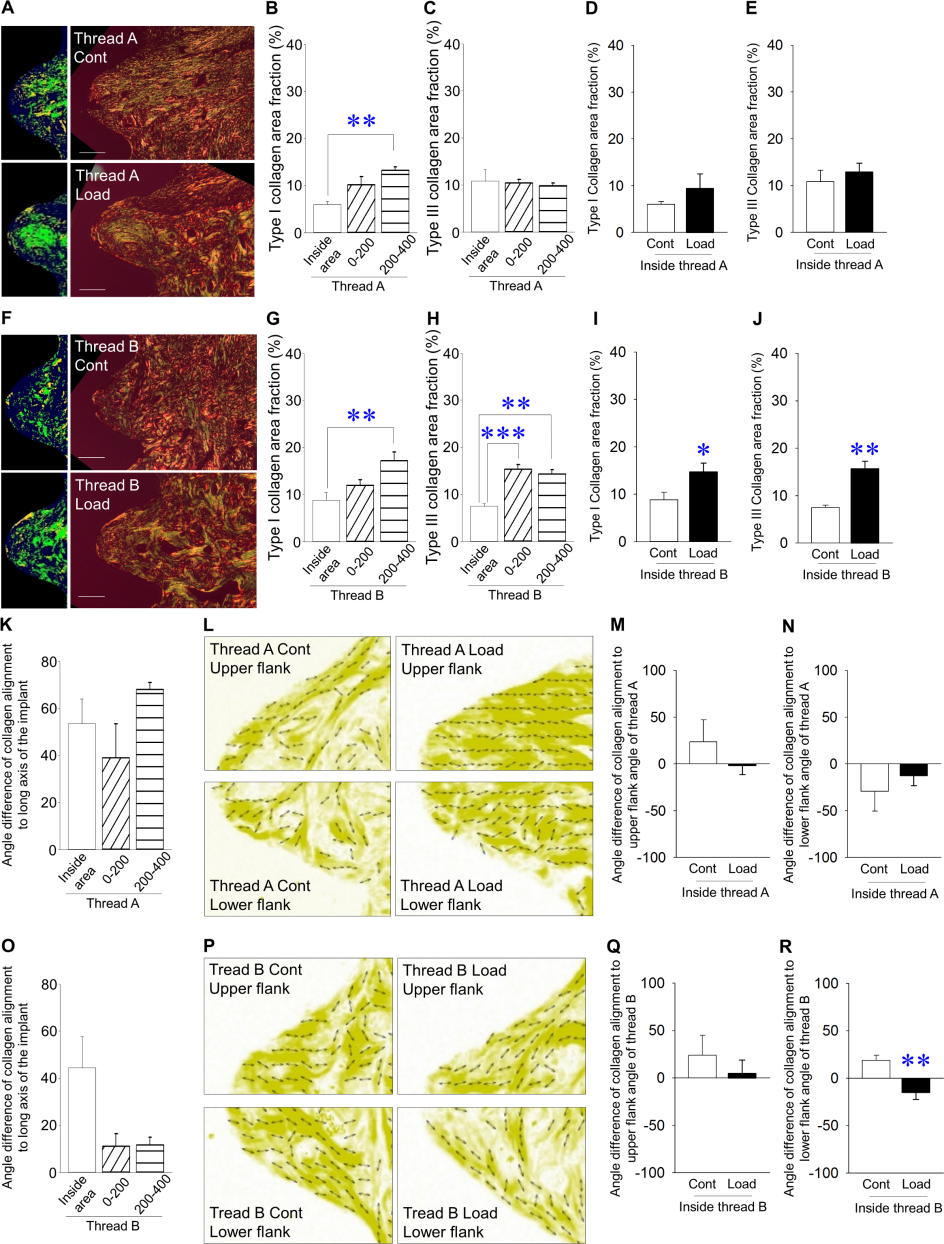
**Effects of mechanical repetitive load on collagen fibers inside and outside area of threads A and B** (A) Representative images of picrosirius red-stained sections with polarized microscopy and emphasized images in thread A (Bar: 100 μm; green and yellow-orange indicate type III and type I collagen, respectively). (B) Type I collagen area fraction was significantly smaller inside thread A vs. outside thread A at 200–400 μm away from implants under non-loaded conditions. (C) Type III collagen area fraction was similar, irrespective of analyzed areas under non-loaded conditions. (D) Type I collagen area fraction was higher in the loading vs. control group, but the difference was not significant. (E) Type III collagen area fraction was significantly higher in the loading vs. control groups. (F) Representative images of picrosirius red-stained sections with polarized microscopy and emphasized image in thread B. (Bar: 100 μm; green and yellow-orange represent type III and type I collagen fibers, respectively) (G) Type I collagen area fraction was smaller inside thread B vs. outside thread B at 200–400 μm away from implants under non-loaded conditions. (H) Type III collagen area fraction was smaller inside thread B vs. outside thread B at 0–200 μm and 200–400 μm away from implants under non-loaded conditions. (I) Type I collagen area fraction was significantly higher in the loading vs. control group. (J) Type III collagen area fraction was significantly higher in the loading vs. control groups. (K) Angle differences of collagen alignment to the long axis of implants were similar among groups under non-loaded conditions. (L) Representative images of the preferential alignment of collagen fibers in thread A (yellow represents collagen fibers and double arrowheads indicate the preferential alignment of collagen fibers). (M) Angle difference of collagen alignment in the upper flank of thread A was similar between groups. (N) Angle difference of collagen alignment in the lower flank of thread A was similar between groups. (O) The angle difference of collagen alignment to the long axis of implants inside thread B was bigger than that outside thread B, but the difference was not statistically significant. (P) Representative images of the preferential alignment of collagen fibers in thread B. (Q) Angle difference of collagen alignment in the upper flank angle of thread B was the same between groups. (R) Angle difference of collagen alignment in the upper flank angle of thread B was significantly smaller in the loading vs. control groups. (Bar: 100μm) (n = 7 each, *p<0.05, **p<0.01).

Representative picrosirius red-stained images using a polarized microscopy for the inside area of thread A (Fig 5A). Under non-loaded conditions, the distribution of type I collagen inside thread A was significantly smaller when compared with that outside thread A at 200–400 μm away from implants (Fig 5B). No change in type III collagen was observed at thread A, regardless of analyzed sites (Fig 5C). Inside thread A, mechanical repetitive load increased the production of type I collagen fibers, but no statistically significant differences were noted (Fig 5D). Mechanical repetitive load significantly increased the production of type III collagen fibers (Fig 5E). Representative picrosirius red-stained images using polarized microscopy for the inside area of thread B (Fig 5F). Under non-loaded conditions, the distribution of type I collagen inside thread B was significantly smaller when compared with that outside thread B at 200–400 μm away from implants (Fig 5G). The distribution of type III collagen inside thread B was significantly smaller when compared with that outside thread B (Fig 5H). Inside thread B, mechanical repetitive load significantly increased the production of both type I and III collagen fibers (Fig 5I and 5J, respectively). Under non-loaded conditions, angle differences of collagen alignment to the long axis of implants were almost same in thread A, irrespective of the distance from implants (Fig 5K). Representative images of the preferential alignment of collagen fibers using a birefringence measurement system in thread A (Fig 5L). Mechanical repetitive loading did not change the angle difference of collagen alignment to the upper and lower flank angles of thread A (Fig 5M and 5N, respectively). Under non-loaded conditions, the angle difference of collagen alignment to the long axis of implants inside thread B appear to be bigger than that outside thread B, but the difference was not statistically significant (Fig 5O). Representative images of preferential alignment of collagen fibers using birefringence measurement system in thread B (Fig 5P). Mechanical repetitive load significantly lessened the angle difference of collagen alignment to the lower flank angle of thread B, but not the upper flank angle of thread B (Fig 5Q and 5R, respectively).

## Discussion

We demonstrated that mechanical repetitive load had different effects on Sema3A production and bone quality around implants between inside threads A and B. We also showed that mechanical repetitive load improves bone quality based on an increased number of osteoblasts and osteocytes, and increased production of type I and III collagen with preferential alignment inside thread B.

Bone remodeling and tooth extraction socket healing takes approximately 6 and 10–14 days in rats, respectively [33, 34], suggesting that about 2 weeks are necessary for healing after tooth extraction. However, our previous study using rat mandible indicated that complete bone healing takes 21–28 days following tooth extraction [26]. Hence, in the present study, a period of 4 weeks was selected in order to allow complete wound healing following tooth extraction. On the other hand, bone integration to implants takes at least 3 weeks in rat maxillae [29]. Combined with bone remodeling after tooth extraction, a 3-week healing period should be appropriate until the initiation of mechanical loads. Thus, mechanical repetitive load does not affect bone healing after implant placement, suggesting that mechanical repetitive load influences bone modeling and remodeling around implants, but not wound healing. Loading conditions such as load magnitude, frequency and cycles are important factors determining bone reactions in response to mechanical loads. In rats, occlusal forces and mastication frequency are 7–11 N and 3–4 Hz, respectively [27, 28]. Thus, 10 N and 3 Hz were used to mimic rat mastication in this study. On the other hand, load cycles remain controversial. Both 36 and 1800 cycles/day has equally resulted in increasing bone mass and formation using an avian loading model [35]. Conversely, tooth contact occurs at 1800 cycles/day during chewing and swallowing in humans [36]. Hence, 1800 cycles/day were selected as a load cycle in this study.

Caution should be exercised when microCT scanning is performed using bone samples including dental implants, as metal artifacts affect analyses of bone structures. A recent study has indicated that a 48-μm exclusion zone from the implant surface was needed to avoid microCT scanning-induced metal artifacts [30]. The authors have inserted titanium implants into long bones, and they used microCT at 16-μm voxel resolution with an energy level of 70 kV [30]. Scanning conditions and materials used in the present study were similar to that study. Hence, the data obtained from microCT analyses should be reliable in this study, as the ROI was defined as the region cross-sectionally surrounding between 50 μm and 550μm away from the implant surface. Our previous studies indicated that mechanical loads significantly increased both bone volume and BMD around dental implants in rabbit long bones [11, 12]. However, in this study, mechanical loads significantly increased BMD around implants without affecting bone volume. Distinct experimental conditions such as animal species and loading protocol may be correlated with the different results between the present and previous studies. In particular, rabbit long bones possesses a small amount of trabecular bone [14], suggesting that stress distribution could differ from rat jaw bones. BMD, which is completely independent of the latest concept of “bone quality” proposed by the NIH, plays an important role in bone mechanical function with bone quality [7]. Thus, increased BMD by mechanical loads around implants may contribute to the long-term stability of bone around implants in clinical situations. In clinical settings, occlusal forces do not increase bone volume around dental implants, supporting the notion that our new finding–increased BMD with no change in bone volume–is a jaw-specific event in response to mechanical loads.

Interestingly, bone quality based on the number of bone cells inside thread B changed markedly under loaded conditions, but no changes were seen in thread A in the present study. A previous study indicated that the thickness of cortical bone in rat maxillae was approximately 400 μm [37]. The thread pitch of the implant was 400 μm in the present study. Thus, bone structures around threads A and B were positioned in the cortical and trabecular bone, respectively. It has been reported that osseous healing between cortical and trabecular bones differs after implant placement in rabbit long bones [38, 39]. No studies separately investigating trabecular and cortical maxillary bones regarding bone reactions after implant placement have been found, regardless of mechanical load. Therefore, the distinct bone reactions in response to mechanical loads in threads A and B may be due to different bone structures (cortical vs. trabecular bone) in maxillary bones.

Our previous studies have demonstrated that bone cells such as osteoblasts and osteocytes are determinant factors of the new concept of “bone quality” [9, 17, 40, 41]. It has been revealed that development of the osteocyte network induced by mechanical loads via dental implants contributes to improved bone quality in rabbit long bones [11–13]. On the other hand, Osteoprotection, which consists of promoted osteoblast differentiation-induced increases in bone formation and osteoclast differentiation suppression-induced decreases in bone resorption, has recently been proposed [24]. Osteoprotection is regulated by Sema3A predominantly produced by osteoblast lineage cells [23]. Of course, no reports have indicated that Sema3A in bone around implants is controlled by mechanical load. Hence, this is the first report showing that mechanical load via implants increased Sema3A production in bone around implants, although this was site-specific. Different stress distribution between the inside of threads A and B, and distinct stress distribution due to bone structure type could be the cause of different bone reactions to mechanical stimuli in terms of Sema3A production. Mechanical repetitive loads contributed to increases in the number of osteoblasts inside thread B, resulting in the development of an osteocyte network, although no changes in Sema3A production occurred inside thread A without increases in the number of osteoblasts and osteocytes. These conflicting findings inside threads A and B indicate that mechanical repetitive load-induced Sema3A production may be correlated with the control of bone quality by regulating the number of osteoblasts and osteocytes around the implant neck. On the other hand, osteoclast numbers did not change under loaded conditions, irrespective of Sema3A production. It has been demonstrated that Sema3A suppressed proliferation and differentiation of osteoclasts [23, 24]. The reasons are unknown, but molecules other than Sema3A induced by mechanical loads via implants may also affect the number of osteoclasts.

Bone is composed of 85–90% collagenous proteins. Type I collagen is the most abundant bone matrix protein produced by osteoblasts [42]. Interestingly, lower production of type I collagen inside thread B under non-loaded conditions was comparable to that outside the thread at 200–400 μm away from implants by mechanical repetitive loads, although no changes occurred in thread A under loaded conditions. The increases in the number of osteoblasts inside thread B could be linked with the increased production of type I collagen fibers by mechanical loads. On the other hand, type III collagen production occurs in early phases of wound healing in many connective tissues, including bone. Until recently, type III collagen was only known as a regulator of type I collagen fibrinogenesis. However, more recently, it has been reported that type III collagen regulates osteoblastogenesis [43], bone repair and maintenance of osteoblast growth [44], and preservation of the osteogenic potential of mesenchymal stem cells [45]. Hence, in this study, increases in type III collagen fibers by mechanical loads could improve bone quality based on the maintenance and improvement of osteoblastogenesis including mesenchymal stem cells.

The preferential alignment of collagen fibers is one of the most important factors determining bone quality. We have demonstrated that mechanical loads have enhanced bone quality around hip implants by improving collagen alignment parallel to the direction of principal stresses [40]. Moreover, we have shown that mechanical repetitive loads also significantly induced the preferential alignment of collagen fibers inside grooves of the implant neck using rabbit long bones [11]. In these studies, thread angles have been demonstrated to be a key factor determining the preferential alignment of collagen fibers. This angle was 60° downwards direction to a plane perpendicular to the long axis of implants in our previous studies [11, 40]. Moreover, according to the International Organization for Standardization 724 and the Japanese Industrial Standards, triangle threads with 60° angle are basically standardized in metric screw threads including dental implants. Indeed, triangle threads with 60° angle are provided for commercial dental implants in clinical settings [46, 47]. Therefore, 60° thread angle along the long axis of the implants was selected in the present study. Under non-loaded conditions, collagen alignment along the long axis of implants was the same between the inside and outside of threads A and B, indicating that preferential alignment of collagen fibers is not linked with bone quality around implants if they do not receive mechanical loads. It has been reported that collagen alignment was also influenced by material directions, regardless of mechanical load [12, 40]. Thus, absolute values of collagen alignment along the long axis of implants between the upper and lower flanks did not change under non-loaded and loaded-conditions in this study (data not shown). Interestingly, mechanical repetitive load significantly induced the preferential alignment of collagen fibers at only the lower flank of thread B, suggesting that preferential alignment of collagen fibers is locally linked with bone quality around implants when mechanical loads are applied. Local changes in collagen alignment by mechanical load may be due to the distinct stress distribution between upper and lower flank of the implant threads [1, 48]. Moreover, mechanical stretches have been demonstrated to induce the preferential alignment of osteoblasts and collagen fibers *in vitro*, suggesting that load-affected osteoblasts and collagen fibers contribute to the adaptation of bone quality [41]. Further studies investigating the short-term effects of mechanical repetitive loads on collagen alignment are required to clarify the adaptation of bone quality around dental implants in response to mechanical loads.

Taken together, the present results show that mechanical repetitive load via implants contributes to improve bone quality and BMD, but not bone quantity (volume) in the jaw bone, although the same responses may not occur when using long bones under loaded conditions in rats. A previous clinical study suggested that the new concept of “bone quality” plays an important role in osseous healing after tooth extraction in the oral cavity in humans [49], which indicates that the new concept of “bone quality” is an important factor in clinical situations. In implant dentistry, therefore, our present findings using the jaw bone contribute to understanding the new concept of “bone quality” based on bone cells and characteristics of collagen fibers with preferential alignment under loaded conditions.

## Conclusions

We demonstrated that mechanical repetitive load had different effects on Sema3A production and bone quality around implants between the inside of threads A and B. We also showed that mechanical repetitive load improves bone quality based on an increased number of osteoblasts and osteocytes, and increased production of type I and III collagen with preferential alignment inside thread B. Mechanical load-induced Sema3A production may be differentially regulated by the types of bone structures (cortical and cancellous bones) or distinct stress distribution, resulting in the control of bone quality around implants in jaw bones.
